# Neurostructural correlates of optimism: Gray matter density in the putamen predicts dispositional optimism in late adolescence

**DOI:** 10.1002/hbm.24888

**Published:** 2019-12-09

**Authors:** Han Lai, Song Wang, Yajun Zhao, Chen Qiu, Qiyong Gong

**Affiliations:** ^1^ Huaxi MR Research Center (HMRRC), Department of Radiology West China Hospital of Sichuan University Chengdu China; ^2^ Department of Radiology Shengjing Hospital of China Medical University Shenyang Liaoning China; ^3^ Psychoradiology Research Unit of Chinese Academy of Medical Sciences (2018RU011) West China Hospital of Sichuan University Chengdu China; ^4^ School of Sociology and Psychology Southwest Minzu University Chengdu China; ^5^ Department of Psychology, The Faculty of Social Science The University of Hong Kong Pokfulam Hong Kong

**Keywords:** optimism, extraversion, magnetic resonance imaging, personality neuroscience, psychoradiology, depression, anxiety

## Abstract

Dispositional optimism reflects one's generalized positive expectancies for future outcomes and plays a crucial role in personal developmental outcomes and health (e.g., counteracting related mental disorders such as depression and anxiety). Increasing evidence has suggested that extraversion is an important personality factor contributing to dispositional optimism. However, less is known about the association between dispositional optimism and brain structure and the role of extraversion in this association. Here, we examined these issues in 231 healthy high school students aged 16 to 20 years (110 males, mean age = 18.48 years, *SD* = 0.54) by estimating regional gray matter density (rGMD) using a voxel‐based morphometry method via structural magnetic resonance imaging. Whole‐brain regression analyses revealed a significant positive correlation between dispositional optimism and the rGMD of the bilateral putamen after adjusting for age, sex, family socioeconomic status (SES), general intelligence, and total gray matter volume (TGMV). Moreover, prediction analyses using fourfold balanced cross‐validation combined with linear regression confirmed a significant connection between dispositional optimism and putamen density after adjusting for age, sex, and family SES. More importantly, subsequent mediation analysis showed that extraversion may account for the association between putamen density and dispositional optimism after adjusting for age, sex, family SES, general intelligence, TGMV, and the other four Big Five personality traits. Taken together, the current study provides new evidence regarding the neurostructural basis underlying dispositional optimism in adolescents and underscores the importance of extraversion as an essential personality factor for dispositional optimism acquisition.

## 1 INTRODUCTION

As a long‐standing research topic in the fields of psychiatry, psychology, and sociology (Bouchard, Carver, Mens, & Scheier, 2018; Scheier & Carver, 2018), dispositional optimism is typically defined as a tendency to hold generalized positive expectancies for future outcomes (Carver & Scheier, 2014; Scheier, Carver, & Bridges, 1994). Dispositional optimism is generally considered a psychological construct that integrates cognition and motivation (Carver & Scheier, 2014; Scheier et al., 1994), including the characteristics of “motivated cognition” (Hughes & Zaki, 2015). Considerable research has shown that dispositional optimism plays an important positive role in personal health and developmental outcomes, which is similar across cultures (Bouchard et al., 2018; Carver & Scheier, 2014; Jiang et al., 2014; Kwok & Gu, 2017; Lai, 2009; Scheier & Carver, 2018; Wong & Fielding, 2007; Yu, Chen, Liu, Yu, & Zhao, 2015; Zhang et al., 2014). For instance, individuals with higher optimism show longer life spans (Lee et al., 2019), lower acute stress‐induced inflammatory effects (Brydon, Walker, Wawrzyniak, Chart, & Steptoe, 2009; Cohen et al., 1999), better mental adaptation under stress (Nes & Segerstrom, 2006), increased life satisfaction (Wong & Lim, 2009), higher work performance (Medlin & Green Jr, 2009), and broader social networks (Andersson, 2012). Individuals with lower optimism, in contrast, report more loneliness late in life (Rius‐Ottenheim et al., 2012), have worse psychological functioning and higher risks of psychiatric disorders following traumatic events (e.g., depression, anxiety, and posttraumatic stress disorder) (Dooley, Fitzgerald, & Giollabhui, 2015; Jakšić, Brajković, Ivezić, Topić, & Jakovljević, 2012; Peleg, Barak, Harel, Rochberg, & Hoofien, 2009; Ramanathan, Wardecker, Slocomb, & Hillary, 2011), and show slower rates of physical recovery after surgery (Mavros et al., 2011). Although behavioral studies on dispositional optimism have achieved substantial progress (Carver & Scheier, 2014; Scheier & Carver, 2018), the neurobiological substrates underlying dispositional optimism are poorly understood, and identifying these substrates may help better understand the nature of optimism and its relations with health and developmental outcomes.

With the rise of personality neuroscience in the past decade (DeYoung, 2010; DeYoung & Gray, 2009), researchers have begun to explore these issues using a magnetic resonance imaging (MRI) approach. Evidence from a limited number of functional MRI (fMRI) studies has suggested that dispositional optimism is mainly related to the functioning of several prefrontal brain regions (e.g., inferior frontal gyrus [IFG], ventromedial prefrontal cortex [VMPFC], and orbital frontal cortex [OFC]), which are involved in complex cognitive and motivational processes (Bangen et al., 2014; Kuzmanovic, Jefferson, & Vogeley, 2016; Ran et al., 2017; Sharot, Korn, & Dolan, 2011; Sharot, Riccardi, Raio, & Phelps, 2007; Wang, Zhao, et al., 2018; Wu et al., 2015). For example, using a task‐based fMRI design, Sharot et al. (2011) observed that individuals with higher dispositional optimism show reduced tracking of undesirable information, which requires processing in the right IFG, than individuals with lower levels. Moreover, evidence from a resting‐state fMRI study revealed that increased dispositional optimism was associated with decreased functional connectivity between the IFG and VMPFC (Ran et al., 2017), which has been shown to be a prominent brain region in reward value processing (Kuzmanovic et al., 2016). Another resting‐state fMRI study further suggested that higher levels of dispositional optimism were associated with lower local spontaneous activity in the OFC (Wang, Zhao, et al., 2018), which serves a role in encoding reward values (O'Doherty, 2004). However, we could still not ascertain which regional variations in human brain morphology were associated with interindividual differences in dispositional optimism, given that the functional and structural correlates of a trait or an ability may not necessarily situate in the same brain areas (Basten, Hilger, & Fiebach, 2015; Kanai & Rees, 2011). Moreover, interindividual differences in personality traits and behaviors have been shown to be deduced from brain morphological metrics, which can be measured with structural MRI (S‐MRI) techniques (DeYoung, 2010; DeYoung & Gray, 2009; Gardini, Cloninger, & Venneri, 2009; Yarkoni, 2015). Hence, S‐MRI is a feasible tool to explore the neurostructural correlates of dispositional optimism.

To our knowledge, only two S‐MRI studies have examined the neuroanatomical substrates of dispositional optimism. Specifically, using a whole‐brain regression analysis based on a voxel‐based morphometry (VBM) approach, Yang, Wei, Wang, and Qiu (2013) reported a positive relation between the thalamus extending to the parahippocampus gyrus volume and dispositional optimism in a sample of 361 healthy young adults aged 17 to 27 years (mean = 19.96, *SD* = 1.29). Furthermore, another study based on a region‐of‐interest (ROI) approach found that higher dispositional optimism was related to a larger OFC volume in a sample of 61 healthy young adults aged 18 to 34 years (mean = 23.23, *SD* = 4) (Dolcos, Hu, Iordan, Moore, & Dolcos, 2016). Notably, these previous findings were obtained from adult participants, and to our knowledge, no study to date has explored the neuroanatomical substrates of dispositional optimism in younger participants, for example, adolescents, who are undergoing significant changes in neural structure and function (Casey, Jones, & Hare, 2008; Paus, 2005). Importantly, dispositional optimism is increasingly recognized to be critical for adolescents' health (e.g., a lower risk of physical and mental disease) and developmental outcomes (e.g., higher subjective well‐being) (Creed, Patton, & Bartrum, 2002; Kwok & Gu, 2017; Lai, 2009; Oreskovic & Goodman, 2013; Puskar, Sereika, Lamb, Tusaie‐Mumford, & Mcguinness, 1999; Wong & Lim, 2009). Therefore, determining the neurostructural basis of dispositional optimism in this period of development is warranted and beneficial as the findings may help identify distinct biomarkers related to dispositional optimism, which may reveal key targeted brain regions for optimism‐promoting interventions to improve adolescents' health and quality of life. Thus, the first goal of the present study was to identify the brain regions in which the gray matter (GM) structure was associated with dispositional optimism in a large group of healthy high school students aged 16 to 20 years (*N* = 231, mean age = 18.48 years, *SD* = 0.54) who are younger and more homogeneous (with respect to age) compared to samples in previous work, providing sufficient statistical power to identify differences (Mackey et al., 2015; Mar, Spreng, & Deyoung, 2013).

Additionally, to better clarify the nature of dispositional optimism, a main goal for personality psychologists is to understand the position of dispositional optimism in the basic human personality construct (e.g., the Big Five model) (Scheier et al., 1994; Sharpe, Martin, & Roth, 2011). In the literature, dispositional optimism has been consistently shown to have a strong positive association with extraversion (Marshall, Wortman, Kusulas, Hervig, & Vickers Jr, 1992; Røysamb & Strype, 2002; Sharpe et al., 2011; Williams, 1992) as these two constructs are conceptually connected. Specifically, as a fundamental trait among the Big Five personality traits, extraversion has the characteristics of positive affection and reward‐motivated behavior (Depue & Collins, 1999; Goldberg, 1990, 1993; Lai et al., 2019; Markon, Krueger, & Watson, 2005), both of which contribute to the formation of optimistic beliefs and an overall positive view of the future (Sharot et al., 2011; Sharot, Guitart‐Masip, Korn, Chowdhury, & Dolan, 2012). Thus, an extrovert would report more positive emotions (DeNeve & Cooper, 1998; Hermes, Hagemann, Naumann, & Walter, 2011; Watson & Clark, 1997) and expect their future outcomes to be more desirable than an introvert (Marshall et al., 1992; Røysamb & Strype, 2002; Sharpe et al., 2011; Williams, 1992). Despite of the conceptually close connections between these two constructs, extraversion is considered a broader, more basic, or superordinate personality trait than dispositional optimism in the hierarchical structure of personality (Costa & McCrae, 1995; Markon, 2009; Markon et al., 2005; Sharpe et al., 2011). According to the perspective of trait hierarchy, personality structure starts from a cluster of root traits and extends downward to generate more specific traits; therefore, higher order traits may influence the development of lower order traits (Costa & McCrae, 1995; Markon, 2009; Markon et al., 2005). Given the conceptual and hierarchical relationships between dispositional optimism and extraversion, extraversion may operate as one of the antecedents for the development of dispositional optimism (Sharpe et al., 2011). Moreover, at the neural level, many studies have revealed a significant association between structures of the brain reward circuitry (e.g., the OFC, VMPFC, and striatum) and extraversion (Canli et al., 2001; Cremers et al., 2011; Grodin & White, 2015; Hermes et al., 2011; Hooker, Verosky, Miyakawa, Knight, & D'Esposito, 2008; Hutcherson, Goldin, Ramel, McRae, & Gross, 2008; Mobbs, Hagan, Azim, Menon, & Reiss, 2005; Rauch et al., 2005; Suslow et al., 2010; Zou, Su, Qi, Zheng, & Wang, 2018). In light of these findings, the second goal of the current study was to test whether the brain regions related to dispositional optimism were linked to extraversion and then to explore the role of extraversion in the association between GM structure and dispositional optimism.

To achieve these goals, we performed S‐MRI scans on participants and assessed their levels of dispositional optimism and extraversion with standard measures. Here, the brain GM structure was estimated using a VBM method (Ashburner & Friston, 2001; Scarpazza & De Simone, 2016). Specifically, we used a VBM approach to assess regional GM density/concentration (rGMD, i.e., the proportion of GM relative to other tissue types in a brain region; Mechelli, Price, Friston, & Ashburner, 2005) as this index is frequently used in developmental studies because maturational changes in GMD in some brain regions (e.g., the frontal and striatum regions) occur at a certain age (Gogtay et al., 2004; Paus, 2005; Sowell et al., 2003; Sowell, Thompson, Tessner, & Toga, 2001), and this index is also often adopted to detect the GM correlates of a personality trait (Omura, Todd Constable, & Canli, 2005; Takeuchi et al., 2011, 2014). Given that an important purpose of this study is to identify prominent brain structures linked to dispositional optimism in late adolescents, we used rGMD as a structural metric. Then, a whole‐brain regression analysis was performed to identify the brain areas related to dispositional optimism. In consideration of previous neuroimaging findings on dispositional optimism, we conjectured that the typical brain regions involved in complex cognitive and motivational processes (e.g., IFG, VMPFC, and OFC) may be associated with dispositional optimism. Moreover, given that the striatum, another prominent structure of the brain reward circuitry (Schultz, 2000), has been found to be closely linked to motivation (Liljeholm & O'Doherty, 2012), we also conjectured that its core structures (e.g., the putamen, caudate nucleus, and nucleus accumbens) may be additional critical neurostructural sites related to dispositional optimism. Next, condition‐by‐covariate interaction analyses (Mosconi et al., 2004; Perrin et al., 2005) were carried out to explore whether sex differences existed in the association of dispositional optimism with rGMD given that some researchers have reported an influence of sex on associations between personality traits and GM structures (Lai et al., 2019; Nostro, Muller, Reid, & Eickhoff, 2017). Finally, correlation analyses and mediation analyses were conducted to probe the associations among GM structure, dispositional optimism, and extraversion. Informed by the existing neurostructural findings on extraversion and the role of extraversion in dispositional optimism, we further expected that some brain regions related to dispositional optimism may be linked to extraversion, and that extraversion may account for the covariations of GM structures with dispositional optimism.

## 2 METHODS

### 2.1 Participants

Two hundred thirty‐four local high school students aged 16 to 20 years (112 males, mean age = 18.6 years, *SD* = 0.78) participated in the study, all of whom were right‐handed native Mandarin Chinese speakers and were recruited from an ongoing neuroimaging project with the purpose of exploring the behavioral and neural substrates underlying social abilities, personality, and academic achievement among adolescents in Chengdu, China (Li et al., 2018; Wang et al., 2017a, 2017b; Wang, Dai, et al., 2018; Wang, Zhao, et al., 2018). All participants were healthy and had no history of psychiatric or neurological diseases according to their records in the student archives from the schools and their self‐reports for two items (i.e., “Have you and your parents ever had any neurological illnesses?” and “Have you and your parents ever had any psychiatric illnesses?”) (Wang et al., 2019). Three participants were excluded from the following analyses as a result of abnormal brain morphological structure (e.g., unusual cysts). Therefore, data from 231 participants aged 16 to 20 years (110 males, mean age = 18.48 years, *SD* = 0.54) were included in the final analyses. This study was in accordance with the approval from the local research ethics committee at West China Hospital of Sichuan University. All participants provided written informed consent before the study.

### 2.2 Behavioral measures

#### 2.2.1 Revised life orientation test (LOT‐R)

We used the LOT‐R (Scheier et al., 1994) to measure an individual's dispositional optimism. The LOT‐R is an unidimensional scale consisting of six items, three of which are worded in positive statements such as “In uncertain times, I usually expect the best,” while the other three of which are worded in negative statements such as “If something can go wrong for me, it will.” The participants were asked to show the degree of their agreement on each item, with a 5‐point rating scale ranging from 1 (strongly disagree) to 5 (strongly agree). After reverse scoring the negatively worded items, we added together the responses for all six items to generate an individual's LOT‐R score, with a higher score suggesting higher dispositional optimism. The Chinese version of the LOT‐R has shown adequate reliability and validity in different populations (Shi, Liu, Wang, & Wang, 2016; Wang, Zhao, et al., 2018; Wang, Liu, Shi, & Wang, 2016; Yang et al., 2013). In our sample, the Cronbach's alpha coefficient for LOT‐R was .74, indicating acceptable internal reliability.

#### 2.2.2 NEO five‐factor inventory (NEO‐FFI)

We used the 12‐item NEO‐FFI‐Extraversion (Costa & McCrae, 1992) to measure an individual's extraversion. To test the specificity of the findings, the four other subscales of the NEO‐FFI (Costa & McCrae, 1992), i.e., neuroticism, conscientiousness, agreeableness and openness, were administered. The participants were asked to show the degree of their agreement on each item, with a 5‐point rating scale ranging from 1 (strongly disagree) to 5 (strongly agree). The Chinese version of the NEO‐FFI has adequate reliability and validity in adolescents and adults (Li et al., 2017; Wang et al., 2017b; Zhang & Huang, 2001). In our sample, the Cronbach's alpha coefficients for the NEO‐FFI subscales ranged between .71 and .81, indicating adequate internal reliability.

#### 2.2.3 Family socioeconomic status (SES)

Considering the substantial association of family SES with dispositional optimism (Heinonen et al., 2006; Renaud, Wrosch, & Scheier, 2018; Robb, Simon, & Wardle, 2009; Taylor & Seeman, 1999) and with GM structures (Brito & Noble, 2014; Jednoróg et al., 2012; Noble, Houston, Kan, & Sowell, 2012; Yaple & Yu, 2019), we assessed individuals' family SES with a subjective SES scale (Adler, Epel, Castellazzo, & Ickovics, 2000) to eliminate possible effects of family SES on dispositional optimism and GM structures. With this scale, the participants were presented with a picture of a ladder with 10 rungs and were required to mark the rung that they believed to correspond to their parents' occupational prestige, income and education (1 = lowest rank; 10 = highest rank) in the local society. The Chinese version of this measure has been indicated to be reliable and valid (Kong et al., 2019; Wang et al., 2017a). Previous studies have shown a close association between subjective SES and physical and psychological health (Adler et al., 2000; Cundiff & Matthews, 2017).

#### 2.2.4 Raven's advanced progressive matrix (RAPM)

In addition, given that general intelligence has been shown to be associated with GM structures (Basten et al., 2015) and extraversion (Wolf & Ackerman, 2005), we evaluated individuals' general intelligence with the RAPM (Raven, 2000) to eliminate possible effects on GM structures and extraversion. The RAPM contains 36 nonverbal items, which required the participants to choose the missing part from each graphical matrix. We totaled the number of correct answers obtained within 30 min as each participant's general intelligence score (Wang, Dai, et al., 2018). In our sample, the Cronbach's alpha coefficient was .83, indicating satisfactory internal reliability.

### 2.3 MRI data acquisition and preprocessing

#### 2.3.1 Data acquisition

The S‐MRI data were collected on a 3.0‐T Trio Erlangen MRI (Siemens, Germany) using a 12‐channel head coil at West China Hospital of Sichuan University, Chengdu, China. We adopted a magnetization‐prepared rapid gradient‐echo sequence to acquire the high‐resolution T1‐weighted anatomical images for each participant. The scanning parameters used in this study were as follows: voxel size, 1 × 1 × 1 mm^3^; flip angle, 9°; matrix size, 256 × 256; slice thickness, 1 mm; 176 slices; echo time, 2.26 ms; inversion time, 900 ms; repetition time, 1,900 ms.

#### 2.3.2 Data preprocessing

After a medical radiologist, who did not know the purpose of this study, visually checked each image, Statistical Parametric Mapping program (SPM12; Welcome Department of Cognitive Neurology, London, UK; http://www.fil.ion.ucl.ac.uk/spm/) was employed to preprocess the S‐MRI data. The following procedure was implemented to determine rGMD (Ashburner, 2015; Mechelli et al., 2005). First, the origin of the images was manually set to the anterior commissure to obtain a better registration. Second, the T1‐weighted anatomical images were segmented into GM, white matter (WM), and cerebrospinal fluid with the new segmentation in SPM12 (Ashburner & Friston, 2005). Third, the GM images were aligned and resampled to 2 × 2 × 2 mm^3^, normalized to a study‐specific template in Montreal Neurological Institute (MNI152) space, and then smoothed with an 8‐mm full‐width at half‐maximum (FWHM) Gaussian kernel using Diffeomorphic Anatomical Registration Through Exponentiated Lie algebra (DARTEL) in SPM12 (Ashburner, 2007). These resulting images (unmodulated) representing the rGMD were used in the subsequent analyses.

### 2.4 Statistical analyses

#### 2.4.1 VBM analyses

To identify brain GM correlates underlying dispositional optimism, a whole‐brain multiple regression analysis was performed in which the rGMD of each voxel in the whole brain was taken as the dependent variable (DV) and the LOT‐R score was taken as the independent variable (IV). Given the potential associations of rGMD with age, sex, family SES, and general intelligence (Frangou, Chitins, & Williams, 2004; Luders et al., 2005; Noble et al., 2012; Sowell et al., 2001, 2003), these items served as covariates in the regression models. In addition, since total GM volume (TGMV, i.e., the total absolute amount of GM) and rGMD are not completely irrelevant and a significant individual difference usually exists in TGMV (Mechelli et al., 2005), we also included TGMV as a covariate as in previous research (Buss, Davis, Muftuler, Head, & Sandman, 2010; Wang et al., 2017a). Then, a condition‐by‐covariate interaction analysis was carried out to examine whether sex differences existed in the association of dispositional optimism with rGMD. In this model, we took sex as a condition, the LOT‐R score as a covariate of interest, and age, family SES, general intelligence, and TGMV as covariates of no interest. The t‐contrasts were employed to check the sex by dispositional optimism interaction effects on the rGMD. An absolute threshold masking of 0.2 according to VBM Tutorial (Ashburner, 2015) was used in the abovementioned analyses to rule out the edge effects between WM and GM (i.e., noise voxels). Moreover, random field theory (RFT), a popular method that considers both peaks and spatial extent via modeling noise as Gaussian random fields (Ashburner & Friston, 2000; Mechelli et al., 2005; Worsley, Evans, Marrett, & Neelin, 1992), was used to correct multiple comparisons. This approach provided significant clusters of voxels at the familywise error (FWE) rate of *p <* .05 (for a *p*‐voxel threshold < .001). The above analyses were performed with SPM12.

#### 2.4.2 Prediction analyses

To investigate whether the identified brain regions whose rGMD was associated with dispositional optimism were stable, we performed prediction analyses using fourfold balanced cross‐validation combined with linear regression, which has been widely used in previous neuroimaging studies (e.g., Cohen et al., 2010; Evans et al., 2015; Kong et al., 2019; Kong, Chen, Xue, Wang, & Liu, 2015; Supekar et al., 2013). These analyses were performed in Matlab R2010a (The MathWorks, Inc., Natick, MA) with the codes used in our previous studies (Wang et al., 2017a; Wang, Dai, et al., 2018; Wang, Zhao, et al., 2018) using the following steps. First, the whole dataset was divided into four folds under the restraint of no significant differences among the distributions in the fourfold data. Second, three folds were employed to establish a linear regression model to predict the fourth fold. Specifically, we extracted each participant's rGMD of the voxels previously identified to be linked to dispositional optimism and then built the linear regression model with the extracted rGMD as the IV and the LOT‐R score as the DV and controlled for variables (i.e., age, sex, and family SES) potentially linked to dispositional optimism (Heinonen et al., 2006; Puskar et al., 2010; Renaud et al., 2018; Robb et al., 2009; Taylor & Seeman, 1999; You, Fung, & Isaacowitz, 2009). Here, general intelligence and TGMV were not included as covariates as no obvious evidence showed associations with dispositional optimism. Thus, a predicted value of dispositional optimism for the fourth fold was calculated and used to compute the correlation between the predicted value and the observed value to obtain *r*_(predicted, observed)_, which represents the predictive ability of the IV on the DV. The second step was repeated four times to obtain the final *r*_(predicted, observed)_ (*r*_final (predicted, observed)_) (i.e., the average of the four *r*_(predicted, observed)_ values). Third, a nonparametric testing method was applied to test the significance of the *r*_final (predicted, observed)_ by generating 5,000 surrogate datasets following the test procedures applied in the previous studies (Cohen et al., 2010; Evans et al., 2015; Kong et al., 2015, 2019; Supekar et al., 2013; Wang et al., 2017a; Wang, Dai, et al., 2018; Wang, Zhao, et al., 2018). Additionally, according to the guidelines proposed by Cohen (1988), a correlation coefficient of .1 indicates a “small” effect size, .3 indicates a “medium” effect size, and .5 indicates a “large” effect size.

#### 2.4.3 Mediation analysis

Next, we investigated the role of extraversion in the association between previously identified GM structures and dispositional optimism. Mediation analysis is a prevalent pathway analysis method usually employed when a strong association exists between an IV and a DV and theoretical or empirically manipulative causal relations exist among research variables (Baron & Kenny, 1986). Given that this method has been applied in numerous studies, especially those based on cross‐sectional or panel designs (Bolandzadeh et al., 2014; Dolcos et al., 2016; Gonzales et al., 2010; Kong et al., 2015, 2019; Mill, Allik, Realo, & Valk, 2009; van Sloten et al., 2016), to statistically depict a potential pathway illustrating how an IV is linked to a DV, we conducted a mediation analysis using the PROCESS macro in SPSS (Hayes & Scharkow, 2013). We assumed that extraversion may explain the association between brain anatomy and dispositional optimism as extraversion may theoretically be one of the antecedents for the development of dispositional optimism (Sharpe et al., 2011).

Specifically, in the mediation model, the DV was the dispositional optimism score, the IV was the rGMD of the voxels previously identified to be linked to dispositional optimism, and the mediator variable (MV) was the extraversion score, and we controlled for age, sex, and family SES, which are potentially linked to dispositional optimism and extraversion (Heinonen et al., 2006; Lucas & Donnellan, 2009; Lynn & Martin, 1997; Puskar et al., 2010; Renaud et al., 2018; Robb et al., 2009; Taylor & Seeman, 1999; Wolf & Ackerman, 2005; You et al., 2009). In addition, general intelligence and TGMV were also included as covariates based on evidence indicating correlations with extraversion (Kunz, Reuter, Axmacher, & Montag, 2017; Wolf & Ackerman, 2005). Based on conventional methodology (Hayes & Scharkow, 2013; MacKinnon, Warsi, & Dwyer, 1995), the indirect effect, i.e., mediation effect, is the product of path *a* (the correlation of the IV with MV) and path *b* (the correlation of the MV with DV after controlling for the IV), or is the difference between path *c* (the correlation of the IV with DV), and path *c*′ (the correlation of the IV with DV after controlling for MV). We used bootstrapping procedures (Pituch, Stapleton, & Kang, 2006), in which we generated a 95% confidence interval (CI) with 5,000 surrogate datasets, to assess the statistical significance of the indirect effect. The indirect effect was significant (*p* < .05) as long as the CI did not contain zero.

## 3 RESULTS

### 3.1 Neurostructural basis of dispositional optimism

The descriptive statistics for dispositional optimism, personality measurements, family SES, and general intelligence are listed in Table 1. The scores of all study variables (except for age) were normally distributed as the absolute skewness and kurtosis values were less than 1 (Marcoulides & Hershberger, 1997). No significant associations of dispositional optimism with age (*r* = −.10, *p* = .115), family SES (*r* = .13, *p* = .055), general intelligence (*r* = −.06, *p* = .370), or TGMV (*r* = −.04, *p* = .584) were found. In addition, no significant sex difference in dispositional optimism (*t*_(229)_ = −1.45, *p* = .145) was identified. We next examined the GM correlates of dispositional optimism.

**Table 1:** Descriptive statistics for age, dispositional optimism, Big Five personality traits, family socioeconomic status (SES), and general intelligence (*N* = 231, 110 males)

Variable	Mean	*SD*	Range	Skewness	Kurtosis
Age	18.48	0.54	16–20	0.50	1.71
Dispositional optimism	22.59	3.01	12–29	−0.36	0.37
Neuroticism	34.03	6.63	15–52	−0.07	−0.10
Extraversion	42.36	6.28	25–58	−0.12	−0.08
Openness	41.23	4.75	31–57	0.28	−0.24
Agreeableness	42.92	4.63	30–57	−0.11	0.17
Conscientiousness	39.86	5.70	22–55	0.10	0.01
Family SES	5.27	1.49	1–9	−0.11	−0.16
General intelligence	24.18	5.68	6–36	−0.26	−0.09

*Note*: Sex and age were self‐reported by each participant, which were consistent with the records (age and sex at birth) in the student archives from the schools.

**Figure 1:**
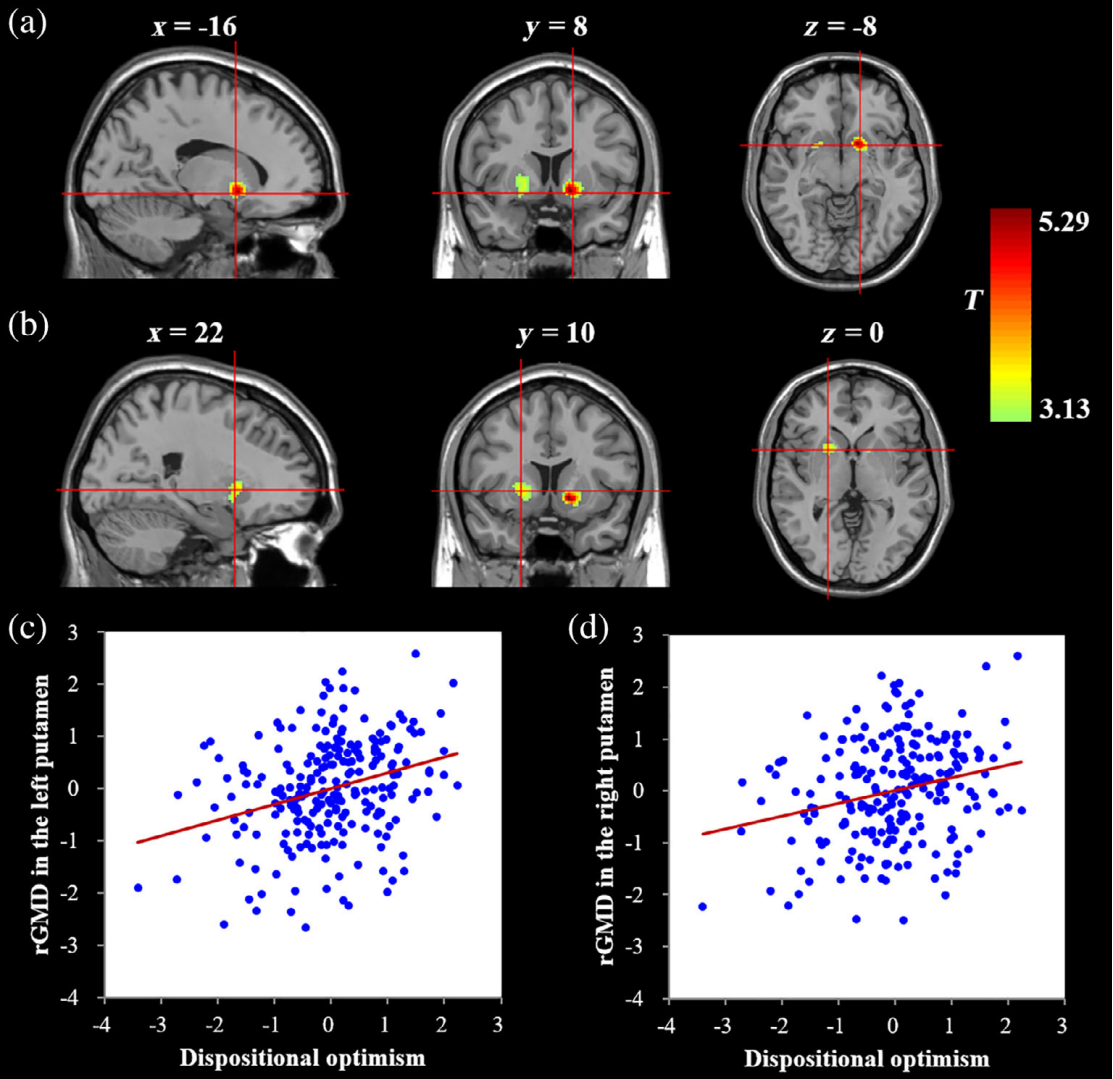
Brain regions related to dispositional optimism. (a) The regional gray matter density (rGMD) of the left putamen is positively associated with dispositional optimism. (b) The rGMD of the right putamen is positively associated with dispositional optimism. (c) A scatterplot showing the correlation between the left putamen and dispositional optimism. (d) A scatterplot showing the correlation between the right putamen and dispositional optimism. Age, sex, family socioeconomic status (SES), general intelligence, and total gray matter volume were adjusted in these analyses

Whole‐brain multiple regression analyses were conducted to detect the brain regions associated with dispositional optimism. Dispositional optimism was found to be positively associated with the rGMD of the bilateral putamen (see Table 2 and Figure 1) after controlling for age, sex, family SES, general intelligence, and TGMV. No other significant cluster was identified. In addition, a condition‐by‐covariate interaction analysis, in which sex was the condition and dispositional optimism was the covariate of interest, was conducted to examine whether the correlation of dispositional optimism with the rGMD varied between males and females. The results revealed no brain region showing the interaction effect of sex by dispositional optimism.

**Table 2:** Brain regions where rGMD was significantly associated with dispositional optimism

Region	Peak MNI coordinate			Peak *T* score	Cluster size (mm^3^)
	*x*	*y*	*z*		
Positive correlation					
Left putamen	−16	8	−8	5.29	1,240
Right putamen	22	10	0	3.67	944

*Note*: The threshold for significant regions was set as follows: *p* < .001 at the voxel level and *p* < .05 at the cluster level, the Gaussian random field approach.

Abbreviations: MNI, Montreal Neurological Institute; rGMD, regional gray matter density.

Then, to investigate the reliability of the connection between the putamen identified from the whole‐brain multiple regression analyses and dispositional optimism, prediction analyses were performed. Dispositional optimism was found to be significantly predicted by the rGMD of the left putamen (*r*_final (predicted, observed)_ = .27, *p* < .001) and the right putamen (*r*_final (predicted, observed)_ = .20, *p* < .001) after adjusting for age, sex, and family SES. Given that highly significant correlations (*r* = .69, *p* < .001) and similar results for the bilateral putamen were found, we used the average rGMD of the bilateral putamen in subsequent analyses.

### 3.2 The role of extraversion

To investigate the role of extraversion in the relationship between the rGMD of the putamen and dispositional optimism, the NEO‐FFI‐Extraversion scale was employed to measure individuals' extraversion. In addition, the other four Big Five personality traits were measured to test the specificity of extraversion. Behaviorally, dispositional optimism was significantly associated with extraversion (*r* = .39, *p* < .001), neuroticism (*r* = −.22, *p* < .001), openness (*r* = .20, *p* = .002), and agreeableness (*r* = .15, *p* = .023) but not with conscientiousness (*r* = .09, *p* = .150). Furthermore, Steiger's *Z* tests found that dispositional optimism was more strongly correlated with extraversion than with the other four Big Five personality traits (Steiger's *Z* tests: *Z*_*s*_ > 2.28, *p*_*s*_ < .05). We next performed a multiple regression analysis to examine the independent impact of each personality trait on dispositional optimism. The results showed that extraversion (*β* = .35, *p* < .001) but not the other four Big Five personality traits (neuroticism: *β* = −.08, *p* = .231; openness: *β* = .10, *p* = .147, agreeableness: *β* = .10, *p* = .121; conscientiousness: *β* = −.03, *p* = .696) can significantly explain the variance in dispositional optimism, suggesting that extraversion has a more crucial association with dispositional optimism.

Then, we explored whether the rGMD of the putamen that was associated with dispositional optimism could be related to extraversion. The results revealed that putamen density was significantly associated with extraversion (*r* = .31, *p* < .001) but not with the other four Big Five personality traits (neuroticism: *r* = −.12, *p* = .067; openness: *r* = .10, *p* = .144, agreeableness: *r* = .02, *p* = .714; conscientiousness: *r* = .03, *p* = .646) after adjusting for age, sex, family SES, general intelligence, and TGMV. Further prediction analyses revealed that individual differences in extraversion can be significantly predicted by the rGMD of the putamen (*r*_final (predicted, observed)_ = .27, *p* < .001) with age, sex, family SES, general intelligence, and TGMV as control variables.

**Figure 2:**
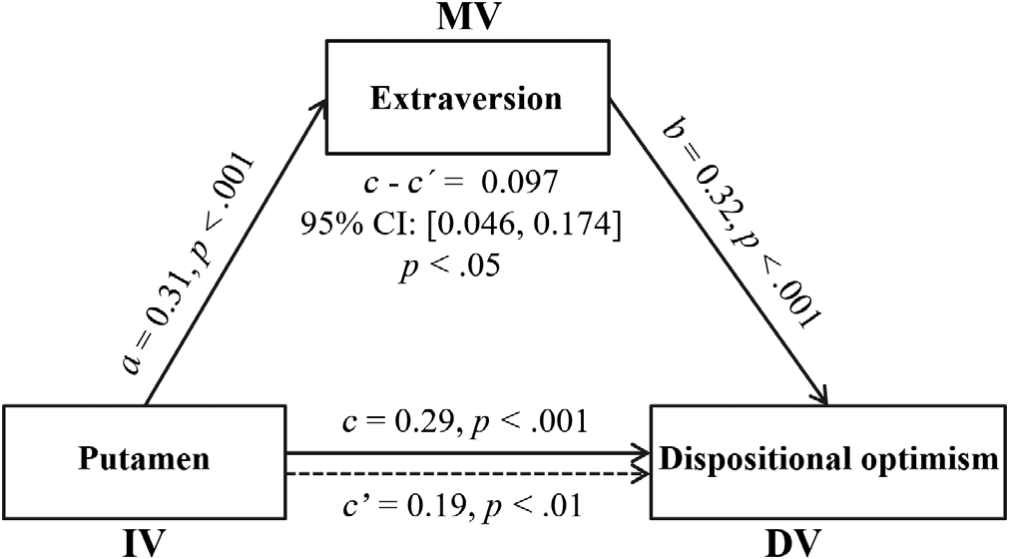
Extraversion explains the association between the regional gray matter density (rGMD) of the putamen and dispositional optimism. The illustration demonstrates that the bilateral putamen affects dispositional optimism though extraversion. All path coefficients are shown as standard regression coefficients (*a*, *b*, *c*, and *c*′), and the indirect effect (*a*×*b* or *c*–*c*′) is significant. Age, sex, family socioeconomic status (SES), general intelligence, and total gray matter volume were adjusted in these analyses

Taken together, the above findings confirmed the close relationships among the rGMD of the putamen, extraversion, and dispositional optimism. To test whether extraversion could explain the relation between the rGMD of the putamen and dispositional optimism, a mediation analysis was performed using age, sex, family SES, general intelligence, and TGMV as covariates. As expected, when extraversion was included as a mediator, the association between the rGMD of the putamen and dispositional optimism was weakened, although the association was still significant (Figure 2). Moreover, bootstrapping procedures demonstrated that extraversion explained the association between the rGMD of the putamen and dispositional optimism (indirect effect = 0.097; 95% CI = [0.046, 0.174], *p* < .05). This result remained significant even when controlling for the other four Big Five personality traits (indirect effect = 0.074; 95% CI = [0.032, 0.145], *p* < .05), indicating the reliability and specificity of the finding.

### 3.3 Supplemental analyses

Given that regional GM volume (rGMV, i.e., the absolute amount of GM within a region; Mechelli et al., 2005) is also a common structural metric used in structural neuroimaging studies (Mechelli et al., 2005), we additionally assessed this index and conducted supplemental analyses to further examine the specific nature of our findings. The detailed methods and findings are described in the Supplemental Material.

## 4 DISCUSSION

The present study aimed to explore the association of GM structure with dispositional optimism and the role of extraversion in this association among healthy adolescents. Whole‐brain multiple regression analyses showed that greater rGMD of the bilateral putamen was significantly linked to higher dispositional optimism. Prediction analyses confirmed the significance of these associations, although the predictive abilities were only small to medium. Moreover, mediation analysis revealed that extraversion could account for the relationship between the rGMD of the putamen and dispositional optimism. Crucially, these results remained significant even when controlling for the nuisance effects of age, sex, family SES, etc., indicating that the abovementioned results were specific to dispositional optimism. Overall, our findings provide new evidence for the neurostructural substrates of dispositional optimism in adolescents and underscore that extraversion may be an essential personality factor for acquiring dispositional optimism.

First, we detected a positive association of the rGMD of the putamen with dispositional optimism. This fits well with the findings showing diminished putamen GM structure in low optimism‐related psychiatric disorders, such as major depression disorder, anxiety disorder, and posttraumatic stress disorder (Bora, Harrison, Davey, Yücel, & Pantelis, 2012; Filipovic et al., 2011; Lu et al., 2016; Parashos, Tupler, Blitchington, & Krishnan, 1998; Yoo et al., 2005). This finding is also in line with a diffusion tensor imaging study with a healthy population reporting an association of optimism with WM connectivity (i.e., the number of WM fiber tracts) between the putamen and frontal regions (Moutsiana, Charpentier, Garrett, Cohen, & Sharot, 2015). As an essential part of reward‐related circuitry, the putamen is considered to be a critical structure related to motivation (Miller, Shankar, Knutson, & McClure, 2014; Mizuno et al., 2008), which is a crucial factor contributing to dispositional optimism (Carver & Scheier, 2014; Scheier et al., 1994). Therefore, we surmised that the finding of the putamen, whose rGMD was linked to dispositional optimism, may reflect the motivational characteristics of dispositional optimism. More concretely, the putamen has been demonstrated to be involved in several motivation‐related processes, for example, history‐based reward value encoding, action selection, and cue‐action‐dependent reward prediction (Elliott, Newman, Longe, & Deakin, 2004; Haruno & Kawato, 2006; Hori, Minamimoto, & Kimura, 2009; Kunimatsu, Maeda, & Hikosaka, 2019; Mizuno et al., 2016; Muranishi et al., 2011; Pessiglione, Seymour, Flandin, Dolan, & Frith, 2006; Schultz, 2000). For example, several studies have shown that the putamen was closely associated with reward value encoding and action selection based on past experience (Kunimatsu et al., 2019; Muranishi et al., 2011), and that these processes, i.e., history‐based reward value updating and action selection, were impaired if the neural activity in the putamen was blocked (Muranishi et al., 2011). Moreover, other studies have shown that the putamen exhibited strong activation in tasks needing the integration of the anticipation of reward with planned actions toward receiving that reward (Elliott et al., 2004; Haruno & Kawato, 2006; Hori et al., 2009). Therefore, the diversity of the rGMD of the putamen probably leads to differences in potential variations within motivational states, which may subsequently affect dispositional optimism levels. When setting expectations for future events, individuals with the greater putamen density may consider future events to be more rewarding based on their past experience and make better action plans to increase the likelihood of achieving those events so that they are more likely to be highly motivated and develop with optimistic expectancies for future events. In sum, our findings suggest the potential pivotal role of the putamen in the development of dispositional optimism. Notably, the present study found that only the putamen was linked to dispositional optimism and failed to identify other brain regions connected to dispositional optimism, such as the IFG, VMPFC, and OFC, which have been reported in previous investigations (Bangen et al., 2014; Dolcos et al., 2016; Kuzmanovic et al., 2016; Ran et al., 2017; Sharot et al., 2007, 2011; Wang, Zhao, et al., 2018). This discrepancy may be due to interstudy differences in brain metrics, sample characteristics and statistical and methodological models. Considering that only the rGMD was employed as a GM morphological indicator in this study, other neurostructural measures (e.g., cortical thickness, surface area, cortical folding, and curvature) should also be utilized in the future to explore the neuroanatomical correlates of dispositional optimism.

More importantly, we found that although the Big Five personality traits (except for conscientiousness) were associated with dispositional optimism, only extraversion could independently predict dispositional optimism. Moreover, extraversion was found to independently explain the association between the rGMD of the putamen and dispositional optimism. These findings indicated that extraversion is a prominent personal resource for acquiring dispositional optimism. Previous neuroimaging studies have discovered that extraversion is significantly related to the activation and morphometry of the putamen (Canli et al., 2001; Hermes et al., 2011; Suslow et al., 2010; Zou et al., 2018). For example, Canli et al. (2001), using a task‐based fMRI approach, observed stronger activation in the putamen in healthy adult extraverts than introverts when viewing positive pictures. Hermes et al. (2011), using an arterial spin labeling perfusion technique, found a lower putamen baseline blood flow in healthy extraverted individuals than in introverted individuals. Furthermore, evidence from a VBM study with healthy adults showed a significant GMV difference in the putamen between the extraverts and introverts (Zou et al., 2018). These findings, together with ours, suggest that the putamen is probably an important region associated with extraversion. In view of the role of the putamen in reward sensitivity (Mizuno et al., 2016), the involvement of the putamen may help individuals perceive more reward value and experience more positive emotions, which contribute to the development of higher levels of extraversion that may further enhance one's motivational state to promote their dispositional optimism levels. This result is also consistent with a physiological finding showing that people who are in a positive state through enhanced dopamine function have more optimistic expectations for future events (Sharot et al., 2012). In brief, our findings confirm that extraversion could serve as a potential mechanism explaining the association of the rGMD of the putamen with dispositional optimism.

In addition, we assessed rGMV and conducted supplemental analyses with this index to further examine the specific nature of our findings. Although we confirmed positive connections between the bilateral putamen and dispositional optimism and the potential role of extraversion in these relations using a ROI approach, no significant associations between rGMV and dispositional optimism were detected in whole‐brain multiple regression analyses, suggesting that rGMD may be a more sensitive structural metric for developmental research (Ramsden et al., 2011; Wang et al., 2017a). Future studies are needed to further explore differences between rGMD and rGMV in neuroimaging applications.

Two potential limitations of the current study should be considered. First, the current study used mediation analysis to explore the role of extraversion in the association between the bilateral putamen density and dispositional optimism based on cross‐sectional data. Although cross‐sectional approaches to mediation may help provide insight into a potential pathway for the development of dispositional optimism to some extent from a theoretical or statistical perspective, drawing any cause‐effect conclusions is difficult. Therefore, our findings should be interpreted with caution, and the directionality of the current findings can be considered only speculative. Future studies with more sophisticated experimental or longitudinal designs are warranted to further examine the causality among the putamen, extraversion, and dispositional optimism. Second, all the standard behavioral measures (i.e., LOT‐R and NEO‐FFI) that we employed depended on self‐reports despite the good reliability and validity of these measures (Li et al., 2017; Wang et al., 2017b; Wang, Zhao, et al., 2018; Yang et al., 2013; Zhang & Huang, 2001). Future studies are encouraged to adopt other approaches (e.g., implicit test or experimental manipulation) to decrease the impact of response bias (e.g., social desirability).

## 5 CONCLUSION

In conclusion, our study provides new evidence for the neurostructural basis underlying dispositional optimism, showing that the bilateral putamen may be a pivotal neuroanatomical site related to dispositional optimism. More importantly, the present study discovered a potential mechanism of extraversion that may explain the association between the putamen and dispositional optimism. Finally, our findings may be helpful for understanding the link between optimism and health and may provide insight into the selection of key targeted brain regions for interventions aiming to improve dispositional optimism levels among adolescents to ameliorate health problems and increase quality of life. In addition, our work may facilitate the progress of psychoradiology (https://radiopaedia.org/articles/psychoradiology), a frontier of radiology aiming at exploring abnormal structural and functional brain changes in psychiatric disorders and also guiding clinical diagnosis and treatment planning decisions in these disorders (Gong, 2020; Huang, Gong, Sweeney, & Biswal, 2019; Lui, Zhou, Sweeney, & Gong, 2016).

## CONFLICT OF INTEREST

The authors declare no competing interests.

## Supporting information


**Appendix S1**. Supporting Information.

## Data Availability

The data and code that support the findings of this study are available from the corresponding author upon reasonable request. The data and code sharing adopted by the authors comply with the requirements of the funding institute and with institutional ethics approval.
