# Supplementary material for: Neurostructural correlates of optimism: Gray matter density in the putamen predicts dispositional optimism in late adolescence
Source: Hum Brain Mapp. 2019 Dec 9;41(6):1459–71. doi: 10.1002/hbm.24888 (PMC7267983; doi:10.1002/hbm.24888)
Supplement: Supplementary file 1 — Appendix S1. Supporting Information. [file HBM-41-1459-s001.docx]

Supplemental analyses

Specifically, we performed another VBM procedure to obtain rGMV (Ashburner, 2010; Mechelli, Price, Friston, & Ashburner, 2005). This procedure differs from the method of determining rGMD in that the GM images were modulated with the Jacobian determinants before they were warped to MNI space and smoothed with an 8-mm FWHM Gaussian kernel. Then, we employed these resulting images representing the rGMV in the whole-brain multiple regression analyses according to the same procedure in the main manuscript. In addition, we took the bilateral putamen as a region of interest (ROI) to examine the association of volume with dispositional optimism and the role of extraversion in this association.

Whole-brain multiple regression analyses did not detect any significant relations between rGMV and dispositional optimism (Gaussian random field theory corrected, *p* < 0.001 at the voxel-level and *p* < 0.05 at the cluster-level) after adjusting for the effect of age, sex, family SES, general intelligence and TGMV. However, the ROI analyses revealed similar positive associations between putamen volume and dispositional optimism (left putamen: *r* = 0.17, *p* = 0.009; right putamen: *r* = 0.13, *p* = 0.044; bilateral putamen: *r* = 0.16, *p* = 0.017). Moreover, as we took the average rGMV of the bilateral putamen for mediation analyses, we found a similar role of extraversion in the relation, where extraversion explained covariation between bilateral putamen volume and dispositional optimism (indirect effect = 0.079; 95% CI = [0.032, 0.151], *p* < 0.05) after adjusting for the effect of age, sex, family SES, general intelligence and TGMV. This result remained significant (indirect effect = 0.059; 95% CI = [0.019, 0.121], *p* < 0.05) after including the other four Big Five personality traits as additional covariates. Therefore, our findings based on rGMV and rGMD are almost consistent, although no significant associations between rGMV and dispositional optimism were detected in whole-brain multiple regression analyses.

References

Ashburner, J. (2010). VBM tutorial. Tech. repWellcome Trust Centre for Neuroimaging, London, UK.

Mechelli, A., Price, C. J., Friston, K. J., & Ashburner, J. (2005). Voxel-based morphometry of the human brain: methods and applications. Current medical imaging reviews, 1(2), 105-113.
